# Botulinum Neurotoxin D Uses Synaptic Vesicle Protein SV2 and Gangliosides as Receptors

**DOI:** 10.1371/journal.ppat.1002008

**Published:** 2011-03-31

**Authors:** Lisheng Peng, William H. Tepp, Eric A. Johnson, Min Dong

**Affiliations:** 1 Department of Microbiology and Molecular Genetics, Harvard Medical School and Division of Neuroscience, New England Primate Research Center, Southborough, Massachusetts, United States of America; 2 Department of Food Microbiology and Toxicology, University of Wisconsin, Madison, Wisconsin, United States of America; The University of Texas-Houston Medical School, United States of America

## Abstract

Botulinum neurotoxins (BoNTs) include seven bacterial toxins (BoNT/A-G) that target presynaptic terminals and act as proteases cleaving proteins required for synaptic vesicle exocytosis. Here we identified synaptic vesicle protein SV2 as the protein receptor for BoNT/D. BoNT/D enters cultured hippocampal neurons via synaptic vesicle recycling and can bind SV2 in brain detergent extracts. BoNT/D failed to bind and enter neurons lacking SV2, which can be rescued by expressing one of the three SV2 isoforms (SV2A/B/C). Localization of SV2 on plasma membranes mediated BoNT/D binding in both neurons and HEK293 cells. Furthermore, chimeric receptors containing the binding sites for BoNT/A and E, two other BoNTs that use SV2 as receptors, failed to mediate the entry of BoNT/D suggesting that BoNT/D binds SV2 via a mechanism distinct from BoNT/A and E. Finally, we demonstrated that gangliosides are essential for the binding and entry of BoNT/D into neurons and for its toxicity *in vivo*, supporting a double-receptor model for this toxin.

## Author Summary

BoNTs are a family of seven bacterial toxins (BoNT/A-G). Among the seven BoNTs, whether BoNT/D uses the same entry pathways and similar receptor-binding strategies as other BoNTs is not known. Previous studies have suggested that BoNT/D does not need a protein receptor nor ganglioside co-receptor, in contrast to all other BoNTs. Here we demonstrate that BoNT/D uses synaptic vesicle protein SV2 as its protein receptor and gangliosides as co-receptor, thus supporting the “double-receptor” model as a central theme for this class of toxins. Furthermore, we found that BoNT/D utilizes a SV2 binding mechanism distinct from BoNT/A and BoNT/E, two other BoNTs that use SV2 as receptors. This indicates that different BoNTs can develop their distinct mechanisms to target a common receptor protein.

## Introduction

Botulinum neurotoxins (BoNTs), one of the six Category A potential bioterrorism agents, are a family of bacterial toxins that cause the fatal disease botulism in humans and animals [1], [2], [3]. These toxins target and enter presynaptic nerve terminals by receptor-mediated endocytosis. Once inside neurons, they act as proteases to cleave host proteins essential for synaptic vesicle exocytosis. Blocking vesicle exocytosis abolishes the release of neurotransmitters from nerve terminals, thus paralyzing muscles, and may cause death due to respiratory failure [1], [4]. The ability of BoNTs to block synaptic vesicle release also provides the basis for their medical applications: local injections of minute amounts of toxin can attenuate neuronal activity in the targeted region which can be beneficial in many medical conditions such as dystonia [5], [6], [7], [8].

BoNTs are classified into seven serotypes (BoNT/A-G) based on their antigenic properties [1], [9]. They share a similar overall domain structure composed of a heavy chain (∼100 kDa) and a light chain (∼50 kDa) connected via a disulfide bond [1], [4], [10]. The heavy chain contains two functional domains: the C-terminal receptor binding domain (HCR, ∼50 kDa) and the N-terminal domain (HN) that mediates the translocation of the toxin light chain (LC) across endosomal membranes. The LCs act as zinc-dependent proteases. The specificity of each toxin LC for host proteins has been well-established: BoNT/B, D, F, and G cleave synaptic vesicle protein synaptobrevin II (Syb, also known as VAMP II) [11], [12], [13], [14], [15], [16]. BoNT/A, E, and C cleave peripheral membrane protein synaptosomal-associated protein of 25 kDa (SNAP-25) [12], [17], [18], [19], [20], [21]. In addition, BoNT/C can also cleave the plasma membrane protein syntaxin (Syx) [22], [23]. These three proteins are known as soluble N-ethylmaleimide sensitive factor attachment protein receptors (SNARE) that form the basic machinery mediating the fusion of synaptic vesicle membranes to plasma membranes [24], [25], [26], [27], [28].

There are two key functional variations among BoNTs: the particular SNARE proteins that their LCs cleave, and the cellular receptors that they use to enter cells. Understanding these key determinants for each BoNT is critical for developing effective strategies to counteract their toxicity and for utilizing them for scientific and therapeutic applications. Thus, it has been a major focus to identify the receptors for each BoNT [29], [30].

The first binding components identified for BoNTs are widely expressed complex forms of gangliosides (polysialiogangliosides, PSG), a family of glycosphingolipids [31], [32], [33], [34], [35], [36]. PSG are abundantly expressed in neurons and their direct interactions with all seven BoNTs have been characterized. Ganglioside-binding sites within each toxin have been proposed and further supported by mutagenesis and crystal structural studies [32], [33], [34], [35], [36], [37], [38], [39], [40], [41], [42], [43], [44], [45], [46]. On the functional level, it has been shown that blocking ganglioside synthesis using chemical inhibitors reduced the binding and entry of BoNT/A and B in cells [47], [48], [49]. Recently, knockout (KO) mice lacking the ability to synthesize PSG have been created. It has been shown that lacking PSG in these KO mice reduced the toxicity of all seven BoNTs at motor nerve terminals using an e*x vivo* phrenic nerve hemi-diaphragm preparation [42], [43], [46], [50]. Furthermore, BoNT/A, B, E, and G failed to bind and enter hippocampal neurons cultured from PSG deficient mice and this defect can be restored using exogenous gangliosides [49], [51]. Finally, mice lacking PSG showed decreased sensitivities to BoNT/A, B, C, and G *in vivo* [49], [52], [53].

In addition to gangliosides, accumulating evidence suggests that there are specific protein receptors for BoNTs and a double-receptor model has been proposed [29], [54]. Previous studies have established two isoforms of synaptic vesicle membrane protein synaptotagmin (Syt) I and II, in conjunction with PSG, as the receptors for BoNT/B and G [35], [46], [49], [55], [56], [57], [58]. Co-crystal structure of BoNT/B bound to Syt II revealed that the toxin binds the membrane adjacent region of Syt [48], [59]. This binding mechanism is shared by BoNT/G, which has the highest sequence similarity to BoNT/B among the seven BoNTs [40], [41], [46], [58].

The protein receptor for BoNT/A and E was subsequently identified as the synaptic vesicle protein SV2 [51], [60], [61]. SV2 contains twelve transmembrane domains and one major luminal domain (the fourth luminal domain, L4) [62], [63], [64], [65]. In contrast to our detailed understanding of BoNT/B-Syt interactions, how BoNT/A and E recognize SV2 at the molecular level remains to be characterized. What have been shown are: 1) Binding of BoNT/A and E are mediated by SV2-L4; 2) BoNT/A can bind SV2-L4, while there is no detectable binding of BoNT/E to recombinant SV2-L4; 3) all three mammalian isoforms of SV2 (SV2A, B, and C) can function as the receptor for BoNT/A, while BoNT/E likely only utilizes SV2A and SV2B; 4) Mutating a conserved N-linked glycosylation site within SV2-L4 (N573Q in SV2A) blocked the entry of BoNT/E and also reduced the entry of BoNT/A into neurons. In addition, it was suggested that BoNT/F, which has the highest sequence similarity to BoNT/E within the seven BoNT-HCRs, also uses SV2 as its receptor [43], [45]. However, functional evidence is still lacking for the role of SV2 in mediating the binding and entry of BoNT/F into neurons.

The remaining serotypes, BoNT/C and BoNT/D, share the highest sequence similarity to each other among the seven BoNTs [9], [66]. Whether these two toxins share the same mode of receptor-recognition with the other five BoNTs remains unsolved. It has been suggested that BoNT/C and BoNT/D do not need protein receptors since treating rat brain synaptosomes with proteases and heating did not diminish toxin binding [53]. It was further suggested that BoNT/D binds phosphatidylethanolamine but not gangliosides, and lacking PSG did not reduce the toxicity of BoNT/D in mice [53]. On the other hand, recent studies have demonstrated that BoNT/D can bind gangliosides and the toxicity of BoNT/D is reduced at phrenic nerve hemi-diaphragm preparations from PSG deficient mice [42].

Here we established that BoNT/D uses SV2 as its protein receptor via a binding-mechanism distinct from BoNT/A and E. We further determined that gangliosides are essential for the binding and entry of BoNT/D into neurons and for its toxicity *in vivo*, thus extending the “double-receptor” model to this toxin and revealing how members of BoNTs converge onto a central theme yet also have their own individual receptor recognition strategies.

## Results

### BoNT/D enters neurons via recycling of synaptic vesicles

**Figure 1:**
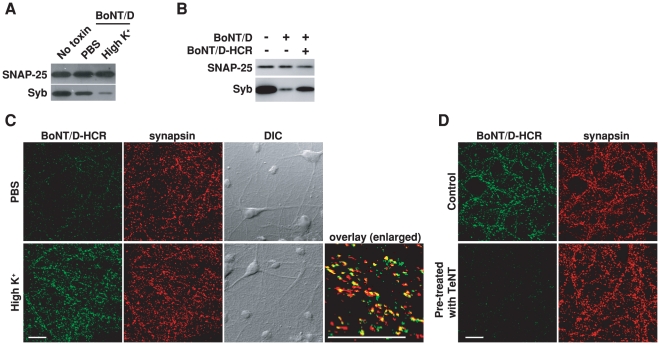
Stimulation of synaptic vesicle recycling increases the binding and entry of BoNT/D into neurons. A) Cultured rat hippocampal neurons were exposed to BoNT/D (100 pM, 5 min) in either resting condition (PBS) or stimulated conditions (high K^+^ buffer: PBS with 56 mM KCl and 1 mM Ca^2+^). Cells were washed and further incubated in toxin-free media for 6 hrs. Cell lysates were subjected to immunoblot analysis. Cells that were not exposed to toxins served as the control (No toxin). SNAP-25 was detected as an internal control for loading of cell lysates. The cleavage of Syb by BoNT/D resulted in loss of Syb immunoblot signals. Stimulation of synaptic vesicle recycling with high K^+^ increased the cleavage of Syb. In all the following experiments, high K^+^ buffer was used to load toxins into neurons using the same procedures described here and cell lysates were subjected to immunoblot analysis, unless otherwise indicated in the Figure Legends. B) Neurons were exposed to BoNT/D (100 pM) with (+) or without (−) the presence of 1 µM BoNT/D-HCR. The presence of BoNT/D-HCR reduced the cleavage of Syb by BoNT/D. C) Neurons were exposed to BoNT/D-HCR (80 nM, 5 min) in either PBS or high K^+^ buffer. Cells were washed, fixed, permeabilized, and subjected to immunostaining analysis. Binding of BoNT/D-HCR was detected using an anti-HA antibody. Synapsin was labeled as a marker for presynaptic terminals. High K^+^ buffer increased the binding of BoNT/D-HCR to presynaptic terminals. The “Overlay” panel is enlarged from the center region of the “high K^+^” sample, showing high degrees of co-localization between BoNT/D-HCR (green) and synapsin (red). The scale bars represent 20 µm in all figures. D) Neurons were pre-treated with TeNT (1 nM, 12 hrs in media) and then tested for the binding of BoNT/D-HCR as described in panel C. Control cells were not exposed to TeNT. Pre-treatment with TeNT prevented the binding of BoNT/D-HCR to neurons.

Exocytosis of synaptic vesicles and subsequent endocytosis of vesicle components is a major membrane recycling event at presynaptic terminals – the target site for BoNTs [67], [68]. Using the cleavage of Syb by BoNT/D as a functional readout for toxin entry, we found that stimulating vesicle exocytosis in cultured rat hippocampal neurons with high levels of potassium solution (high K^+^ buffer) increased Syb cleavage as compared to resting conditions (Figure 1A). We next constructed the receptor binding domain of BoNT/D (BoNT/D-HCR) fused with a HA tag in order to directly assay the binding of toxins to cell surfaces, since there were no suitable antibodies available for BoNT/D detection. This recombinant BoNT/D-HCR is capable of competing with BoNT/D for binding receptors as it reduced the cleavage of Syb by BoNT/D (Figure 1B). We found that high K^+^ buffer increased the binding of BoNT/D-HCR to neurons (Figure 1C). Binding occurs mainly at presynaptic terminals as shown by high degrees of co-localization between BoNT/D-HCR and the presynaptic marker synapsin (Figure 1C, overlay). Moreover, treating neurons with tetanus neurotoxin (TeNT), which cleaves Syb and blocks synaptic vesicle exocytosis [11], blocked the binding of BoNT/D-HCR to neurons (Figure 1D). Together, these data suggest that BoNT/D enters neurons through recycling of synaptic vesicles.

### SV2 is a synaptic vesicle protein binding to BoNT/D-HCR

**Figure 2:**
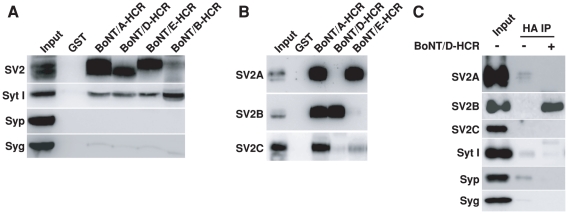
BoNT/D-HCR can pull-down and co-immunoprecipitate SV2 from rat brain detergent extracts. A) The HCRs of BoNT/A, D, E, and B were purified as GST-fusion proteins, immobilized on beads (500 µg), and incubated with 1.5 ml rat brain detergent (Triton X-100) extracts (2 mg/ml, 1 hr at 4°C). GST protein was assayed in parallel as a control. Bound materials were analyzed by immunoblot analysis. BoNT/A, D and E pulled-down SV2 and low levels of Syt I, while BoNT/B pulled-down Syt I. B) Bound materials from HCRs of BoNT/A, D, and E as described in panel A were analyzed using polyclonal antibodies that recognize SV2A, B, or C, respectively. BoNT/A-HCR pulled-down all three isoforms. BoNT/D-HCR pulled-down SV2B, while BoNT/E-HCR pulled-down SV2A. C) HA-tagged BoNT/D-HCR (100 nM) was incubated with brain detergent extracts (0.5 ml, 1 hr at 4°C). Co-immunoprecipitation assays were carried out using a monoclonal anti-HA antibody (HA IP). BoNT/D-HCR co-immunoprecipitated SV2B, but not other vesicle proteins.

We next purified BoNT/D-HCR as a glutathione S-transferase (GST) fusion protein and used it to pull-down interacting proteins from rat brain detergent (Triton X-100) extracts. Bound materials were subjected to immunoblot analysis using antibodies for major synaptic vesicle membrane proteins [68], [69]. The HCRs of BoNT/A, E, B, as well as GST protein alone, were assayed in parallel as controls. BoNT/D-HCR pulled-down significant amounts of vesicle protein SV2 and low levels of Syt I, but not other vesicle proteins such as synaptophysin (Syp) or synaptogyrin I (Syg), in a similar manner to the HCRs of BoNT/A and E (Figure 2A). BoNT/A and E are known to use SV2 as their receptor and the low levels of bound Syt I might be due to the association of Syt I with SV2 as previously characterized [70], [71], [72], [73], [74]. Consistently, the HCR of BoNT/B, which uses Syt I/II as its receptors, pulled-down Syt I but not SV2 (Figure 2A). These data suggest that BoNT/D-HCR can bind SV2.

SV2 shown in Figure 2A was detected using an antibody that recognizes all three mammalian SV2 isoforms. We noticed that the molecular weight of SV2 pulled-down by BoNT/D-HCR and BoNT/E-HCR appears to be different (Figure 2A). Therefore, we further analyzed the bound materials using antibodies specific for each SV2 isoform (Figure 2B). While BoNT/A-HCR pulled-down all three SV2 isoforms, BoNT/D-HCR and BoNT/E-HCR showed clear preferences: BoNT/D-HCR for SV2B (which is of lower molecular weight than SV2A and C), and BoNT/E-HCR for SV2A, respectively. We note that although BoNT/E-HCR did not pull-down detectable levels of SV2B, we have previously demonstrated that SV2B can function as the receptor to mediate the binding and entry of BoNT/E in neurons [51]. The likely explanation for these apparently contradictory results is that the pull-down assay using detergent-solubilized materials may only preserve the strongest binding interactions, while the neuronal surface may provide an optimal environment for SV2-BoNT/E interactions. Similarly, the preference of BoNT/D-HCR for SV2B does not exclude other SV2 isoforms as its receptors on neuronal surfaces, but suggests that BoNT/D-HCR may have the highest binding affinity to SV2B under our assay conditions.

In addition, we also carried out co-immunoprecipitation assays using a HA antibody to immunoprecipitate soluble BoNT/D-HCR incubated with brain detergent extracts (Figure 2C). Immunoprecipitated materials were analyzed using antibodies against different synaptic vesicle proteins. SV2B is the only one co-immunoprecipitated with BoNT/D-HCR at significant levels (Figure 2C); further confirming BoNT/D-SV2 interactions.

### SV2 is required for the binding and entry of BoNT/D into neurons

**Figure 3:**
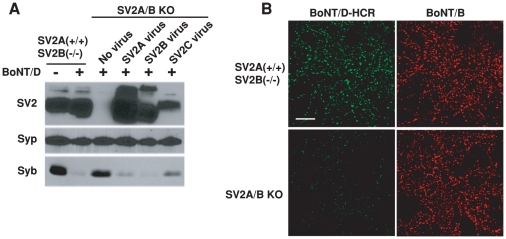
SV2 is essential for the binding and entry of BoNT/D into neurons. A) SV2A/B double KO mice were generated by breeding mice with the genotype of SV2A(+/−)SV2B(−/−). Thus, SV2A(+/+)SV2B(−/−) mice from the same litter as SV2A/B double KO mice served as the control. Hippocampal neurons cultured from these mice were exposed to BoNT/D (100 pM). Cell lysates were subjected to immunoblot analysis using antibodies for SV2, Syp (as a loading control), and Syb. SV2 A/B KO neurons infected with lentiviruses expressing SV2A, B or C were also assayed in parallel. BoNT/D failed to enter SV2 A/B KO neurons as evidenced by the lack of Syb cleavage. Expression of SV2A, B or C restored the entry of BoNT/D. B) SV2A(+/+)SV2B(−/−) and SV2A/B KO neurons were exposed to BoNT/D-HCR (80 nM) and BoNT/B (20 nM) in high K^+^ buffer for 5 min. Cells were subjected to immunostaining analysis. BoNT/B can bind both types of neurons. BoNT/D-HCR failed to bind SV2A/B KO neurons.

To determine whether SV2 plays a role for the binding and entry of BoNT/D, we used hippocampal neurons cultured from SV2A/B double KO mice as a cell model. We have previously found that hippocampal neurons mainly express two of the three SV2 isoforms – SV2A and SV2B, thus SV2A/B KO neurons can serve as a loss-of-function model [51], [60]. We found that BoNT/D failed to enter SV2A/B KO neurons, as demonstrated by the lack of Syb cleavage (Figure 3A). Furthermore, expressing SV2A, B or C in SV2A/B KO neurons via lentiviral infection restored the entry of BoNT/D and resulted in the cleavage of Syb (Figure 3A). These results demonstrated that SV2 is essential for the functional entry of BoNT/D into neurons, and all three isoforms of SV2 can mediate the entry of BoNT/D.

We next examined whether SV2 is required for the binding of BoNT/D to neurons. As shown in Figure 3B, BoNT/D-HCR failed to bind SV2A/B KO neurons. BoNT/B served as an internal control, which bound to both control and SV2A/B KO neurons, demonstrating that loss of BoNT/D-HCR binding is not due to any potential defects in the vesicle recycling process in SV2A/B KO neurons. Furthermore, binding of BoNT/D-HCR was also observed for a subpopulation of SV2A/B KO neurons that still express SV2C and bound BoNT/D-HCR largely co-localizes with endogenous SV2C (Figure S1). Together, these results indicate that SV2 likely mediates the binding of BoNT/D to neurons.

### The binding mechanism of BoNT/D to SV2 is distinct from BoNT/A and BoNT/E

**Figure 4:**
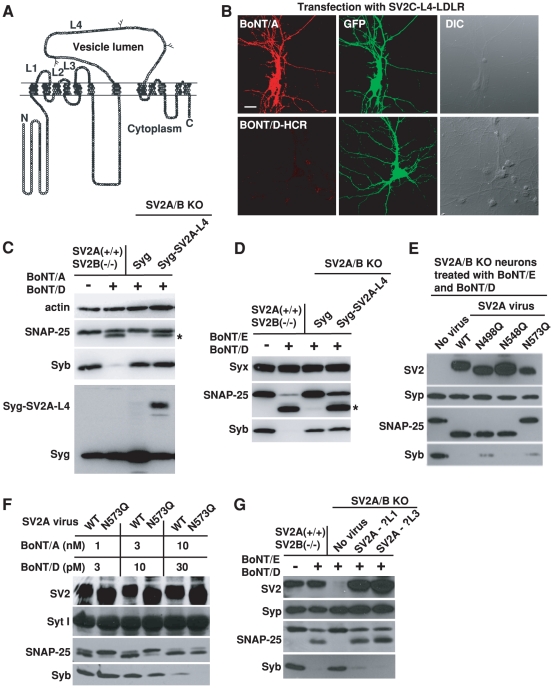
BoNT/D utilizes a SV2-binding mechanism distinct from BoNT/A and E. A) Schematic drawing of SV2. Filled circles indicate conserved residues in all SV2 isoforms; gray circles are residues conserved in two SV2 isoforms; and open circles represent non-conserved residues. Three glycosylation sites within the L4 domain are also indicated. B) SV2A/B KO neurons were transfected with a chimeric receptor containing the SV2C-L4 at the N-terminus of the transmembrane and cytosolic domain of LDL receptor (SV2C-L4-LDLR, [51]). Cells were exposed to either BoNT/A (20 nM) or BoNT/D-HCR (80 nM) for 20 min in culture media, washed and fixed for immunostaining analysis. Transfected cells were marked by co-expressed GFP. SV2C-L4-LDLR mediated the binding of BoNT/A, but failed to mediate the binding of BoNT/D-HCR. C) Chimeric protein Syg-SV2A-L4 was constructed by inserting SV2A-L4 into the second luminal domain of Syg. Syg and Syg-SV2A-L4 were expressed in SV2A/B KO neurons via lentiviral infection. Cells were exposed to BoNT/A (10 nM) and BoNT/D (100 pM). Actin serves as a loading control. Expression of Syg and Syg-SV2A-L4 was confirmed using a polyclonal anti-Syg antibody. Cleavage of SNAP-25 by BoNT/A yielded a smaller fragment that is indicated by an asterisk. Syg-SV2A-L4 mediated the entry of BoNT/A as indicated by the cleavage of SNAP-25, but failed to mediate the entry of BoNT/D as shown by the lack of Syb cleavage. D) Experiments were carried out as described in panel C, except that neurons were exposed to BoNT/E (200 pM) and BoNT/D (100 pM). Cleavage of SNAP-25 by BoNT/E yielded a smaller fragment that is indicated by an asterisk. Syx was used as a loading control. Syg-SV2A-L4 mediated the functional entry of BoNT/E, but not BoNT/D. E) Three glycosylation sites within the SV2A-L4 domain were abolished by site-directed mutagenesis, respectively. These mutants were expressed in SV2 A/B KO neurons via lentiviral infection. Neurons were exposed to BoNT/D (100 pM) and BoNT/E (200 pM). Syp serves as a loading control. Mutation N573Q abolished the entry of BoNT/E. Entry of BoNT/D was not affected by abolishing any one of the three glycosylation sites. F) SV2A/B KO neurons were infected with lentiviruses that express either WT SV2A or SV2A N573Q mutant. Cells were exposed to BoNT/A and BoNT/D at indicated concentrations (5 min in high K^+^ buffer, 12 hrs incubation in media afterwards). Syt I serves as a loading control. Mutation N573Q reduced the cleavage of SNAP-25 by BoNT/A at low toxin concentrations (1 and 3 nM). Cleavage of Syb by BoNT/D is similar at all toxin concentrations between neurons expressing WT SV2A and N573Q mutant. G) SV2A mutants harboring disrupted luminal domain 1 (SV2A-ΔL1) or L3 (SV2A-ΔL3) were expressed in SV2A/B KO neurons via lentiviral infection. Neurons were exposed to BoNT/E (100 pM) and BoNT/D (100 pM). Disrupting L1 or L3 domain did not reduce the functional entry of either BoNT/E or BoNT/D.

SV2 has only one major extracellular domain (luminal domain) with significant length (SV2-L4, ∼130 amino acids, Figure 4A). We previously demonstrated that BoNT/A can bind recombinant SV2-L4 fragments directly [60], while BoNT/E requires glycosylation at a particular site within the SV2-L4 domain [51]. We next carried out a series of studies to determine whether BoNT/D shares SV2-binding mechanisms with either BoNT/A or BoNT/E.

In previous studies, we have constructed chimeric receptors containing SV2-L4 plus the transmembrane and cytoplasmic domains of low density lipoprotein receptor (LDLR) [51]. Once expressed in SV2A/B KO neurons, these chimeric receptors (SV2-L4-LDLR) were able to mediate the binding of BoNT/A (Figure 4B, Figure S2, [51]) and BoNT/E [51], but failed to mediate the binding of BoNT/D-HCR (Figure 4B, Figure S2).

To address the concern that SV2-L4-LDLR does not localize to synaptic vesicles, we next inserted SV2A-L4 into the luminal domain of another multiple membrane spanning synaptic vesicle protein Syg. Once expressed in SV2A/B KO neurons, this chimeric protein (Syg-SV2A-L4) mediated the entry of BoNT/A (Figure 4C) and BoNT/E (Figure 4D) into neurons at a comparable efficiency to endogenous SV2A expressed in control neurons, as indicated by the similar levels of SNAP-25 cleavage. In contrast, Syg-SV2A-L4 failed to mediate the entry of BoNT/D as shown by the lack of Syb cleavage (Figures 4C–D). These data suggest that the SV2-L4 domain, expressed in chimeric receptors, can provide a binding site for BoNT/A and E, but it is not sufficient for BoNT/D.

Because glycosylation at the third site within the SV2-L4 (N573 in SV2A) has been shown to be essential for the entry of BoNT/E and also can enhance the entry of BoNT/A at low toxin concentrations [51], we next examined whether BoNT/D shares this requirement. Three mutant forms of SV2A that harbor point mutations at each N-linked glycosylation consensus sequence (N498Q, N548Q, N573Q), respectively, were expressed in SV2A/B KO neurons. As we previous reported, N573Q mutation completely blocked the entry of BoNT/E and protected SNAP-25 (Figure 4E). In contrast, none of the mutants blocked the entry of BoNT/D (Figure 4E). Furthermore, N573Q did not affect the entry of BoNT/D when we reduce BoNT/D concentrations from 100 pM (Figure 4E) to 30 and 10 pM (Figure 4F). As a control, we confirmed our previous finding that N573Q mutation reduced BoNT/A entry at low toxin concentrations (3 and 1 nM, Figure 4F). These data again suggest that BoNT/D has a SV2-binding mechanism distinct from BoNT/A and BoNT/E. In addition, these studies also demonstrated that the SV2A(N573Q) mutant is able to mediate the entry of toxins, providing strong evidence that mutating the third glycosylation site in SV2A does not affect the function and localization of SV2, but rather specifically abolishes the receptor function for BoNT/E and reduces the binding affinity for BoNT/A.

Besides L4, SV2 has two other short luminal domains (L1: ∼15 amino acids; L3: ∼20 amino acids, Figure 4A). To test whether these two minor domains play any roles for BoNT/D, we constructed two mutant forms of SV2A by deleting the middle portions of L1 and L3 (residue 196-200 of L1 and 321-331 of L3), respectively. Both mutants, when expressed in SV2 A/B KO neurons, were able to restore the entry of BoNT/D and BoNT/E (Figure 4G), indicating these two short luminal domains are unlikely participants in providing the binding site for BoNT/D or BoNT/E.

### Localization of SV2C on plasma membranes reconstituted the binding site for BoNT/D-HCR on the surface of neurons and non-neuronal cells

**Figure 5:**
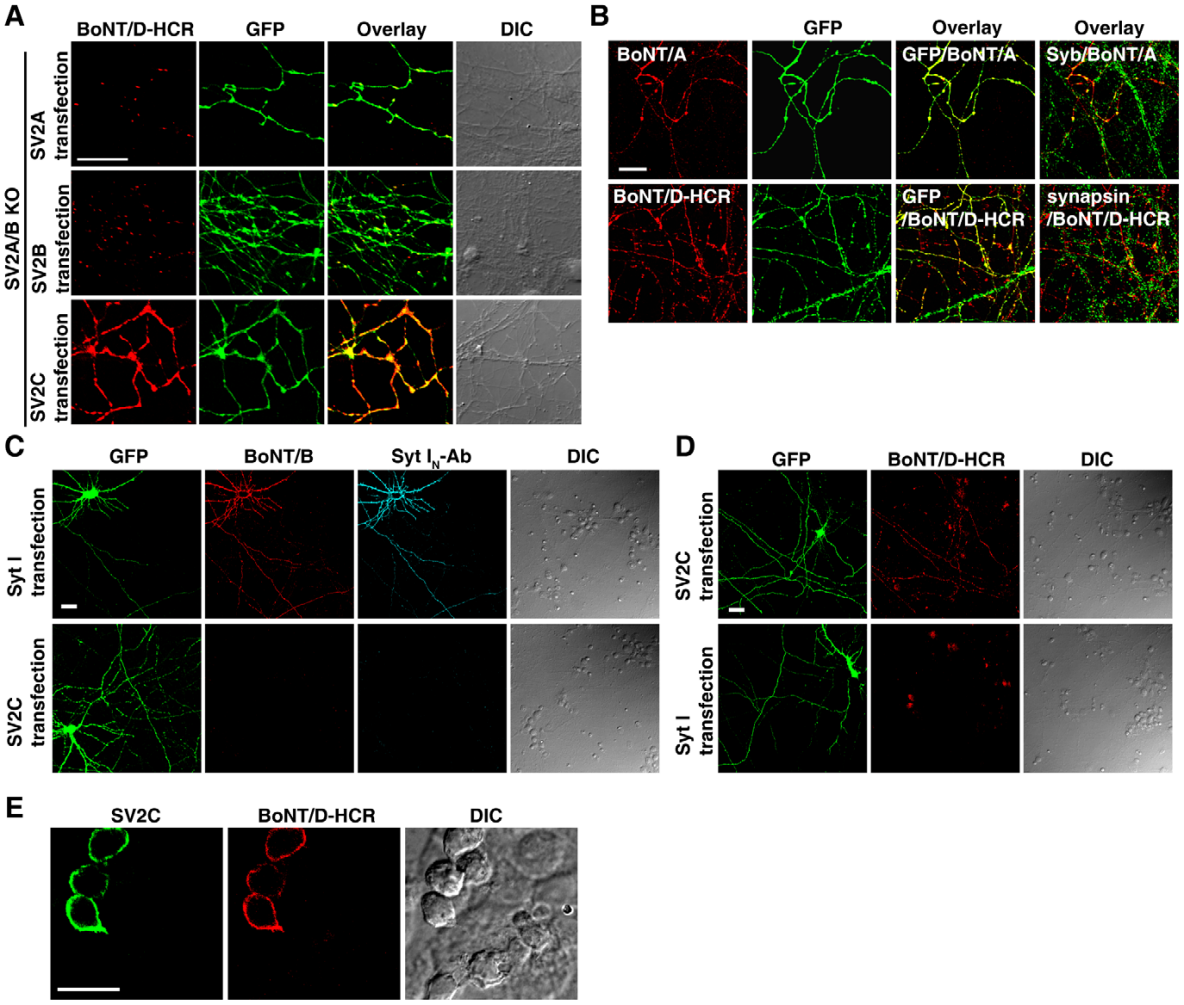
Localization of SV2C to plasma membranes mediated binding of BoNT/D-HCR to the cell surface in both neurons and HEK293FT cells. A) SV2A/B KO neurons were transfected with SV2A, B, or C. Transfected cells were marked by co-expressed GFP. Cells were exposed to BoNT/D-HCR (80 nM, 5 min in high K^+^ buffer), washed, fixed, and permeabilized for immunostaining. Binding of BoNT/D-HCR was observed as puncta along processes in neurons transfected with SV2A (upper panel) or SV2B (middle panel). Binding of BoNT/D-HCR to SV2C-transfected neurons appears to be continuous along neuronal processes (lower panel). B) SV2A/B KO neurons were transfected with SV2C. Cells were exposed to BoNT/A (20 nM, upper panel) or BoNT/D-HCR (80 nM, lower panel) for 10 min in cold media (incubation on ice). Cells were then washed in cold media and fixed. Immunostaining was first performed without permeabilizing cells to detect the surface binding of BoNT/A or BoNT/D-HCR. Cells were subsequently permeabilized and immunostaining was further carried out to label neuronal processes using an anti-GFP antibody and presynaptic terminals using antibodies for Syb (upper panel) or synapsin (lower panel). Transfection of SV2C resulted in the binding of BoNT/A (upper panel) and BoNT/D-HCR (lower panel) to neuronal surface at both presynaptic terminals (co-localized with Syb (green) or synapsin (green)) as well as regions outside the synapse. C) Cultured rat hippocampal neurons were transfected with either Syt I (upper panel) or SV2C (lower panel). Neurons were exposed to BoNT/B (20 nM) and an antibody that recognizes the luminal domain of Syt I (Syt I_N_-Ab, 1∶200) for 10 min in cold media, washed, and fixed. Immunostaining was first performed without permeabilizing cells to detect the surface binding of BoNT/B and Syt I_N_-Ab. Cells were subsequently permeabilized and immunostaining was carried out using an anti-GFP antibody. Transfection of Syt I, but not SV2C, resulted in the binding of both BoNT/B and Syt I_N_-Ab to neuronal surfaces. D) Experiments were carried out as described in panel C, except that neurons were exposed to BoNT/D-HCR (80 nM). Expression of SV2C, but not Syt I, mediated the binding of BoNT/D-HCR to neuronal surfaces. E) HEK293FT cells were transfected with SV2C and exposed to BoNT/D-HCR (80 nM, 30 min in media at 37°C). Immunostaining was first carried out without permeabilizing cells to detect BoNT/D-HCR. Cells were subsequently permeabilized and immunostaining was carried out using a polyclonal anti-SV2C antibody. Expression of SV2C mediated the binding of BoNT/D-HCR to the surface of HEK293FT cells.

Whether SV2 can provide the binding site for BoNT/D on cell surfaces is a key question in establishing it as a receptor for BoNT/D. SV2 luminal domains are the only regions that can be transiently exposed to the outside of cells during vesicle recycling. The finding that LDLR- or Syg-based chimeric receptors failed to mediate the entry of BoNT/D did not exclude the L4 domain as the toxin binding site, especially considering that the L4 domain in SV2 is anchored to membranes through both N- and C-terminal transmembrane domains (Figure 4A), yet these membrane adjacent regions are disrupted in chimeric receptors. Unfortunately, our attempts to include the transmembrane domains of SV2 in different chimeric receptors, as well as various mutations within the L4 domains and the L4 domain deletion all resulted in mis-folded proteins that are not expressed/trafficked in cells, suggesting a rigid requirement for a specific conformation within the L4 domain. In order to examine whether SV2 provided the binding site for BoNT/D, we have to find a way to present SV2 luminal domains onto cell surfaces in their native conformation. The solution comes from a new observation we made when examining the binding of BoNT/D-HCR to SV2A/B KO neurons transfected with different SV2 isoforms (Figure 5A).

SV2 normally resides on synaptic vesicles. As expected, transfecting SV2A or SV2B restored the binding of BoNT/D-HCR to SV2A/B KO neurons in a puncta pattern (Figure 5A, upper and middle panels), suggesting that binding occurs at presynaptic terminals. To our surprise, binding of BoNT/D-HCR to SV2C-transfected neurons showed a continuous binding pattern along neuronal processes (Figure 5A, lower panel). The likely explanation is that a significant portion of SV2C localizes onto plasma membranes and mediates the binding of BoNT/D-HCR to regions outside of synapses.

To confirm the localization of SV2C to plasma membranes, we tested the binding of BoNT/A to SV2C-transfected SV2A/B KO neurons under a low temperature condition, which stops membrane trafficking and only allows the binding to occur at cell surfaces. Furthermore, surface-bound toxins were detected via immunostaining without permeabilizing cells. Under these assay conditions, we detected the binding of BoNT/A to SV2C-transfected neurons in a continuous pattern along neuronal processes (Figure 5B, upper panel). BoNT/A is known to bind SV2C-L4 [60], [61], thus this result demonstrates that SV2C is located on cell surfaces with its luminal domains exposed to the outside of cells. In addition, we subsequently permeabilized these cells and detected synaptic marker Syb (Figure 5B, overlay Syb/BoNT/A). We found that Syb distributed along BoNT/A-bound neuronal processes in a puncta pattern, indicating that these processes are axons harboring presynaptic terminals and also demonstrating that BoNT/A binding occurs at both presynaptic terminals and regions outside of synapses. Under the same assay conditions, we observed robust binding of BoNT/D-HCR to SV2C-transfected neurons (Figure 5B, lower panel) at both presynaptic terminals (labeled by synapsin) and regions outside of synapses, demonstrating that SV2C mediates the binding of BoNT/D-HCR to cell surfaces.

This low temperature surface-binding assay allows us to examine toxin binding even in WT neurons. Using this assay, we found that the receptor for BoNT/B, Syt I, also has a significant portion localized on plasma membranes when over-expressed in rat neurons, as demonstrated by the surface binding of BoNT/B and the binding of Syt I_N_ Ab that recognizes the N-terminus of Syt I luminal domain (Figure 5C, upper panel). Under the same assay conditions, we did not detect the binding of BoNT/B or Syt I_N_ Ab to SV2C-transfected neurons (Figure 5C, lower panel). Consistently, BoNT/D-HCR binds to SV2C-transfected rat neurons (Figure 5D, upper panel), but not to Syt I-transfected neurons (Figure 5D, lower panel), demonstrating the specificity of BoNT/B and BoNT/D-HCR in recognizing their respective receptors under our assay conditions.

Finally, we expressed SV2C in non-neuronal HEK293FT cells. Transfected cells were exposed to BoNT/D-HCR and immunostaining was first carried out without permeabilizing cells to detect the surface binding of BoNT/D-HCR. Cells were subsequently permeabilized to confirm the expression of SV2C using a polyclonal SV2C antibody. As shown in Figure 5E, expression of SV2C mediated the binding of BoNT/D-HCR to the surfaces of HEK293FT cells. Furthermore, the polyclonal SV2C antibody, which recognizes the N-terminal cytoplasmic domain of SV2C, failed to stain SV2C in transfected cells without permeabilizing cells (Figure S3), suggesting that SV2C maintains the correct membrane topology on the surface of HEK293FT cells. Together, the experiments described in this section demonstrate that SV2 functions as a receptor providing the binding site for BoNT/D on cell surfaces.

### PSG are required for the binding and entry of BoNT/D into neurons

**Figure 6:**
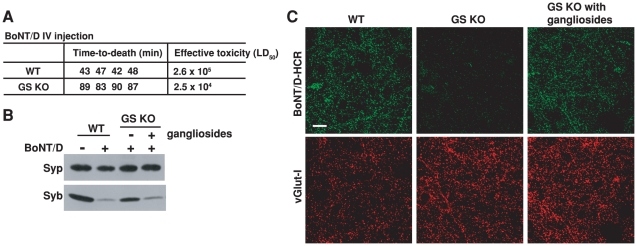
PSG are essential for the binding and entry of BoNT/D into neurons. A) The sensitivity of four pairs of WT and GS KO mice to BoNT/D was assayed using a rapid-time-to-death assay [57]. When injected with the same amount of toxins, all four GS KO mice survived longer than WT mice, corresponding to a 10-fold reduction of effective toxicity *in vivo*. B) Neurons cultured from GS KO mice, with (+) or without (−) incubation with exogenous gangliosides (250 µg/ml ganglioside mixture, 12 hrs in media), were exposed to BoNT/D (100 pM). WT neurons from the same littermates were assayed in parallel. BoNT/D failed to enter GS KO neurons as evidenced by the lack of cleavage of Syb; the entry was restored by adding exogenous gangliosides. C) Neurons cultured from GS KO, with (right panel) or without (middle panel) pre-incubation with exogenous gangliosides, were exposed to BoNT/D-HCR (80 nM) for 5 min in high K^+^ buffer. WT neurons were assayed in parallel (left panel). Cells were washed, fixed, and subject to immunostaining analysis. Vesicular glutamate transporter I (vGlut-I) was labeled as a marker for presynaptic terminals. BoNT/D-HCR failed to bind GS KO neurons and the binding was restored by loading exogenous gangliosides to cell membranes.

We next determined whether BoNT/D requires gangliosides as co-receptors. We note that a previous study concluded that PSG are not required for BoNT/D binding and entry based on a KO mouse line lacking GM3 synthase, which only depletes a- and b- series, but not o-series PSG. O-series PSG are not abundant in WT neurons; however, it has been shown that their levels are significantly elevated in GM3 KO neurons [75]. Thus, we assessed the role of PSG using a different KO mouse line lacking the gene encoding GM2/GD2 synthase (GS KO), an enzyme required for synthesis of all major PSG [76]. Using a well-established rapid-time-to-death assay, we examined the sensitivity of KO mice versus their wild type (WT) littermates to BoNT/D. The assay was conducted by injecting a large amount of toxins (10^5^–10^6^ mean lethal doses, LD_50_) into mice that resulted in death within 30 min to 1 hour. Within this range of toxin concentrations, the effective toxicity *in vivo* can be estimated based on how long the mice survive using a standard curve [57], [77]. When injected with the same amount of BoNT/D, the KO mice survived significantly longer than WT mice (Figure 6A). The effective toxicity in KO mice was reduced to only 10% of the level in WT mice (Figure 6A), demonstrating that PSG are essential for the toxicity of BoNT/D *in vivo*.

We next assayed whether reduced toxicity of BoNT/D in GS KO is due to the decrease of BoNT/D entry into neurons. Using hippocampal neurons cultured from GS KO mice as a cell model, we found that lacking gangliosides reduced the entry of BoNT/D into neurons as evidenced by the reduction of Syb cleavage (Figure 6B). Furthermore, the functional entry of BoNT/D was restored by loading exogenous gangliosides into the cell membrane (Figure 6B).

The next question is whether PSG are required for the neuronal binding step of the toxin action. To directly examine this, we tested the binding of BoNT/D-HCR to hippocampal neurons cultured from GS KO mice and their WT littermates. Binding of BoNT/D-HCR was abolished in GS KO neurons (Figure 6C, middle panel), and it was restored by loading exogenous gangliosides onto the cell membrane (Figure 6C, right panel), demonstrating that gangliosides are required for the binding of BoNT/D to neurons. Together, these studies provided critical evidence at both animal and cellular levels for the conclusion that PSG are essential co-receptors for BoNT/D.

### Entry of BoNT/C and BoNT/F into hippocampal neurons requires PSG but not SV2

Among the seven BoNTs, BoNT/C has the highest sequence similarity to BoNT/D, while BoNT/F has the highest sequence similarity to BoNT/E within the seven BoNT-HCRs [9]. We next assessed whether BoNT/C and BoNT/F share the same requirement for receptor-recognition with BoNT/D or BoNT/E, taking advantage of available PSG and SV2 KO mouse lines.

**Figure 7:**
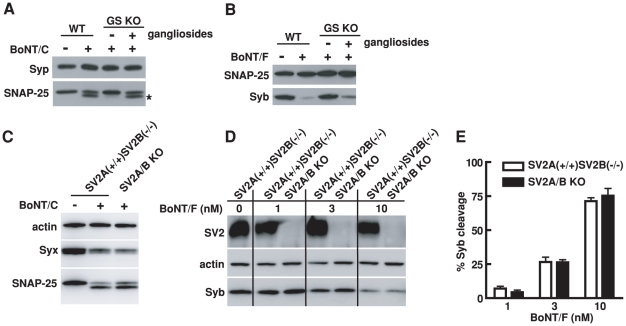
Entry of BoNT/C and BoNT/F into hippocampal neurons requires PSG but not SV2. A) Experiments were carried out as described in Figure 6B, except that neurons were exposed to BoNT/C (10 nM). Cleavage of SNAP-25 by BoNT/C yielded a smaller fragment that is indicated by an asterisk. BoNT/C failed to enter GS KO neurons as shown by the lack of cleavage of SNAP-25, and exogenous gangliosides restored the entry of BoNT/C. Syp serves as a loading control. B) Experiments were carried out as described in panel A, except that neurons were exposed to BoNT/F (10 nM). BoNT/F failed to enter GS KO neurons as evidenced by the lack of cleavage of Syb; the entry was restored by adding exogenous gangliosides. SNAP-25 serves as a loading control. C) Hippocampal neurons cultured from SV2A/B KO mice and their SV2A(+/+)SV2B(−/−) littermates were exposed to BoNT/C (10 nM). In addition to SNAP-25, the second target protein of BoNT/C, Syx, was also detected. Actin serves as a loading control. The cleavage of Syx by BoNT/C resulted in loss of Syx immunoblot signals. Similar degrees of Syx and SNAP-25 cleavage by BoNT/C were observed in SV2A/B KO neurons as compared to control neurons. D) Hippocampal neurons cultured from SV2A/B KO mice and their SV2A(+/+)SV2B(−/−) littermates were exposed to a titration of BoNT/F at indicated concentrations. Lacking SV2 in hippocampal neurons did not significantly reduce the entry of BoNT/F into neurons, as shown by similar levels of Syb cleavage at each toxin concentration between SV2A/B KO neurons and control SV2A(+/+)SV2B(−/−) neurons. E) Degrees of Syb cleavage by BoNT/F assayed in panel D were quantified by densitometry and normalized to the levels of actin. Error bars represent standard deviation (N = 4).

We found that lacking PSG abolished the functional entry of BoNT/C, as shown by the lack of cleavage of SNAP-25 (Figure 7A). Entry was restored by adding exogenous gangliosides to cell membranes (Figure 7A). Similarly, lacking PSG reduced the entry of BoNT/F, as evidenced by the decreased cleavage of Syb (Figure 7B). Loading gangliosides into cell membranes restored the entry of BoNT/F (Figure 7B). These results are consistent with previous studies demonstrating that gangliosides can bind BoNT/C and F, and are essential for their toxicity in mice and in phrenic nerve hemi-diaphragm preparations [43], [45], [53]. Our studies provided further cellular evidence to establish gangliosides as a shared co-receptor for all seven BoNTs.

Lacking SV2, on the other hand, did not significantly reduce the cleavage of SNAP-25 and Syx by BoNT/C in cultured hippocampal neurons as compared to the control neurons that still express SV2A (Figure 7C), suggesting that BoNT/C does not share the same protein receptor requirement as BoNT/D in hippocampal neurons. We also found that the absence of SV2 did not reduce the sensitivity of hippocampal neurons to BoNT/F compared to control neurons that still express SV2A, as evidenced by the similar levels of Syb cleavage at all toxin concentrations examined (Figure 7D,E). Although many questions remain to be determined such as whether other SV2 isoforms play a role in BoNT/F entry, whether SV2 mediates BoNT/F entry into other types of neurons and whether other proteins can compensate the loss of SV2 for BoNT/F, it is clear that BoNT/F does not share the same receptor-binding mechanism with BoNT/E in hippocampal neurons.

## Discussion

To identify the receptor for BoNT/D, we first determined that BoNT/D uses synaptic vesicle recycling to enter neurons. Using BoNT/D-HCR as bait, we identified synaptic vesicle protein SV2 as the toxin binding protein. Utilizing hippocampal neurons cultured from SV2A/B KO mice, we demonstrated that SV2 is essential for the binding and entry of BoNT/D into neurons and all three isoforms of SV2 can mediate the binding and entry of BoNT/D. Our key finding is that localization of over-expressed SV2C onto plasma membranes mediated the binding of BoNT/D to cell surfaces in both neurons and HEK293 cells, suggesting that the luminal domains of SV2 provide the binding site for BoNT/D and demonstrating that other synaptic vesicle proteins are not required. Together, these data establish SV2 as the protein receptor for BoNT/D.

Interestingly, BoNT/D appears to have a SV2-recognition strategy distinct from BoNT/A and BoNT/E. First, SV2-L4 domain expressed in LDLR-based or Syg-based chimeric proteins can function as the receptor for BoNT/A and E, but failed to mediate the entry of BoNT/D. Second, N573Q mutation that abolished a glycosylation site within the SV2A-L4 domain blocked the entry of BoNT/E and also reduced the entry of BoNT/A, but has no significant effect on the entry of BoNT/D. These data suggest that BoNT/D has a distinct SV2-binding mechanism that has yet to be understood. In fact, we still do not understand whether BoNT/A and BoNT/E share similar mechanisms recognizing SV2 at the molecular level. The finding that BoNT/D also uses SV2 as a receptor, together with recent progress solving the crystal structures of BoNTs and BoNT-HCRs [39], [42], [44], [78], [79], provided an opportunity for comparative studies in order to understand the molecular and structural basis for seemingly diverse SV2-binding mechanisms utilized by different BoNTs.

Among the seven BoNTs, BoNT/B and G display the highest similarity and they share Syt I/II as their receptors [9], [58]. BoNT/A and E are fairly close to each other and they share SV2 as the receptor [9]. The finding that BoNT/D also uses SV2 as the receptor, on the other hand, may not be explained by sequence similarity especially considering that the binding mechanism appears to be different from BoNT/A and E. This surprising convergence may suggest that SV2 possesses certain characteristics/functions that make it an attractive receptor candidate. One possibility is that the complex glycan structure in SV2 may facilitate the binding of toxins. It is also interesting to note that Syt is known to function as the Ca^2+^ sensor for triggering vesicle release and also plays an important role for maintaining the rate of synaptic vesicle endocytosis [80], [81], [82], [83], [84]. Although the function of SV2 has not been established, recent studies showed that lacking SV2 results in elevated Ca^2+^ levels in the presynaptic terminals and also reduces the rate of compensatory membrane retrieval after synaptic vesicle release [85]. Moreover, it has been shown that SV2 associates with Syt and may regulate the endocytosis of Syt [70], [74], [82]. The roles of Syt and SV2 in Ca^2+^ signaling and compensatory endocytosis, two critical functions in a vesicle cycle, may provide strategic reasons for toxins to exploit them as receptors to target recycling synaptic vesicles.

Using BoNT/A as a specific SV2 luminal domain probe, we showed that significant portions of SV2C localize onto plasma membranes when over-expressed in neurons. This is likely due to over-expression of exogenous proteins since it was not observed for endogenous SV2C (Figure S1). This phenomenon was also seen for Syt I. It has been proposed that the plasma membrane is a default destination for Syt I and its sorting to synaptic vesicles requires endocytotic sorting adaptors that could be overwhelmed by over-expressed Syt I [86]. It remains to be seen whether a similar mechanism causes the localization of over-expressed SV2C on plasma membranes.

In addition to protein receptors, we also examined the role of PSG for the binding and entry of BoNT/D in neurons utilizing GS KO mice. We showed that these ganglioside deficient mice are less sensitive to BoNT/D *in vivo*. We further showed that BoNT/D cannot bind and enter neurons cultured from GS KO mice; binding and entry can be restored by loading exogenous gangliosides to cell membranes. We extended these studies to BoNT/C and BoNT/F, and demonstrated that PSG are required for the functional entry of both toxins in cultured neurons. Together, these studies contribute to the growing body of evidence that PSG are a shared binding platform for all seven BoNTs.

Finally, we note that a natural chimeric toxin composed of the light chain of BoNT/C and the receptor binding domain of BoNT/D has been tested in patients [87], [88]. This toxin is designated as BoNT/C-D, but has also been marketed as a subtype of BoNT/C [9], [89]. Its receptor binding domain is identical to BoNT/D. Our studies indicate that this toxin targets neurons by recognizing SV2 and gangliosides.

## Materials and Methods

### Ethics statement

This study was carried out in strict accordance with the recommendations in the Guide for the Care and Use of Laboratory Animals of the National Institutes of Health. The protocol was approved by the Standing Committee on Animals of Harvard Medical School (Permit Number: 04619). All efforts were made to minimize suffering of animals.

### Antibodies, mouse lines, toxins and other materials

Mouse monoclonal antibodies for Syb (Cl 69.1), Syt I (Syt I_N_ Ab: Cl 604.4; Syt I cytoplasmic domain Cl 41.1), SV2 (pan-SV2), synaptophysin (Cl 7.2), syntaxin (HPC-1) and SNAP-25 (Cl 71.2) were generously provided by E. Chapman (Madison, WI). Rabbit polyclonal antibodies for BoNT/A and BoNT/B were described previously [57], [60]. Rabbit polyclonal antibodies for SV2A, B, C, and Syg were generously provided by R. Janz (Houston, TX) and were described previously [65], [90], [91]. The following antibodies were purchased from indicated vendors: mouse monoclonal anti-HA (16B12, Covance); rabbit polyclonal anti-synapsin and guinea pig anti-vesicular glutamate transporter I (vGlut-I, Millipore); chicken polyclonal anti-GFP and mouse monoclonal anti-actin (Abcam).

GM2/GD2 synthase KO mice have been previously described [76] and were obtained from the Consortium for Functional Glycomics (Grant number: GM62116). The SV2A/B knockout mice were described previously [90] and were generously provided by R. Janz.

Bovine brain gangliosides were purchased from Matreya LLC (PA). TeNT was purchased from List Biological Lab (CA). BoNT/A (Hall-A), BoNT/B (Okra), BoNT/C (Brazil), BoNT/D (D1873), BoNT/E (Alaska) and BoNT/F (Langeland) were purified in E. Johnson's lab from indicated strains.

### cDNA, constructs and protein purification

Rat SV2A/B/C and Syg cDNAs were described previously [62], [63], [64], [65] and were generously provided by R. Janz. Rat Syt I cDNA were generously provided by T.C. Sudhof (Palo Alto, CA).

Lox-Syn-Syn lentivirus vector [92] was used for all constructs expressing exogenous proteins in neurons. This vector contains two separate neuronal-specific promoters (synapsin promoter). One promoter controls expression of indicated proteins and the other controls expression of EGFP.

LDLR-based chimeric receptors were generated by fusing the L4 domains of each SV2 isoform (residues 468–595 in SV2A, 410–539 in SV2B, 453–580 in SV2C) to the N-terminus of a fragment encoding the transmembrane and cytosolic domain of human LDLR-2 (residues 788-860) as described previously [51]. Syg-based chimeric receptor was constructed by inserting the SV2A-L4 domain between residue 140 (L) and 141 (N) within the second luminal domain of Syg. Deletion mutants SV2A-ΔL1 and SV2A-ΔL3 were generated by replacing residues 196-200 of L1 and 321–331 of L3 with a peptide sequence derived from the first eleven amino acids of rat Syt I [93]. This sequence can be recognized by Syt I_N_-Ab, which we found only recognizes rat but not mouse Syt I [51], thus serving as a tag. Point mutations at N-glycosylation sites of SV2A have been described previously [51].

The cDNA encoding the HCRs of BoNT/A and BoNT/B were generously provided by J. Barbieri (Milwaukee, WI) and were previously described [73]. The cDNA encoding the HCRs of BoNT/D (residues 859–1276, GenBank: CAA38175.1) and BoNT/E (residues 820–1252 GenBank: X62683.1) were synthesized by Geneart Inc. (Germany) with codon optimized for *E. Coli* expression. They were subcloned into pGEX4T vector for expression as GST fusion proteins. In addition, BoNT/D-HCR was also subcloned into pET-28 vector, with a HA-tag (YPYDVPDYA) fused to its N-terminus. This HA-tagged BoNT/D-HCR was purified as N-terminal tagged His_6_-fusion proteins. Both GST-fusion and His_6_-fusion proteins were purified as previously described [94], [95], except that the induction conditions were changed to 16°C overnight with 0.25 mM IPTG.

### Brain detergent extracts, GST pull-down assay and co-immunoprecipitation

Rat brain detergent extracts were prepared by homogenizing one fresh dissected adult rat brain in 15 ml 320 mM sucrose buffer, followed by a centrifugation at 5000 rpm for 2 min at 4°C in a Sorvall SS-34 rotor. Supernatants were collected and centrifuged at 11,000 rpm for 12 min using the same rotor. The pellet was collected and solubilized for 30 min in 15 ml Tris-buffered saline (TBS: 20 mM Tris, 150 mM NaCl) plus 2% of Triton X-100 and a cocktail of protease inhibitors (Roche, CA). Samples were subsequently centrifuged at 17,000 rpm for 20 min in a Sorvall SS-34 rotor to remove the insoluble materials. The final brain detergent extracts yielded ∼2 mg/ml proteins.

GST pull-down assays were carried out using 500 µg GST fusion proteins or GST protein immobilized on glutathione-Sepharose beads, mixed with 1.5 ml rat brain detergent extracts for 1 hr at 4°C. Beads were washed three times with the washing buffer (TBS plus 0.5% Triton X-100). Ten percent of bound proteins were subjected to SDS-PAGE and immunoblot analysis following standard western blot procedures using the enhanced chemiluminescence (ECL) method (Pierce). ‘Input’ corresponds to 0.5% of total brain extracts incubated with each HCR protein.

Co-immunoprecipitation experiments were carried out by first incubating 100 nM HA-tagged BoNT/D-HCR with 0.5 ml rat brain detergent extracts for 1 hr at 4°C, and then after the addition of monoclonal anti-HA antibody (4 µl) incubating for a further 1 hr. Protein G Fast Flow beads (50 µl, GE Bioscience) were then added and incubated for additional 1 hr. The beads were washed three times in the washing buffer (TBS plus 0.5% Triton X-100). Bound proteins were analyzed by immunoblot analysis.

### Neuron cultures, transfection and lentiviral infection

Rat hippocampal neurons were prepared from E18-19 embryos. Mouse hippocampal neurons were prepared from P1 mice. Dissected hippocampi were dissociated with papain following manufacture instructions (Worthington Biochemical, NJ). Cells were plated on poly-D-lysine coated glass coverslips and cultured in Neurobasal medium supplemented with B-27 (2%) and Glutamax (Invitrogen). Experiments were carried out generally using DIV (days in vitro) 12–18 neurons. Transfection of neurons was carried out using Lipofectamine 2000 (Invitrogen) at DIV5. Lentiviral particles were produced by HEK293FT (Invitrogen) cells co-transfected with the virus packaging vectors (VSV-G and Δ8.9) as described previously [92]. Viruses were added to neurons at DIV5.

### Binding and entry of toxins into neurons, surface binding assays and toxin binding to HEK293FT cells

The control buffer (PBS) used in Figure 1A contains (mM: NaCl 140, KCl 3, KH_2_PO_4_ 1.5, Na_2_HPO_4_ 8, MgCl_2_ 0.5). High K^+^ buffer is the same as the control buffer but adjusted to 56 mM KCl and 87 mM NaCl plus 1 mM CaCl_2_. In general, the binding of BoNT/D-HCR to neurons was assayed by incubating neurons with 80 nM BoNT/D-HCR for 5 min in high K^+^ buffers at 37°C. Cells were washed three times. Immunostaining was carried out by fixing cells with 4% paraformaldehyde, permeabilized with 0.3% Triton in PBS solution, and incubated with indicated primary antibodies for 1 hr at room temperature, followed by the incubation with secondary antibodies conjugated with Alexa dyes (Invitrogen, CA) for 1 hr at room temperature. Images were collected using a Leica TCS SP confocal microscope using a 40x oil objective.

Surface binding assays described in Figure 5B–D were carried out by first incubating neurons in cold media (4°C, 5 min), and then exposing them to indicated reagents in cold media on ice for 10 min. Cells were washed and fixed. Immunostaining was first carried out without the permeabilization step to detect the surface binding of BoNT/D-HCR, BoNT/A, BoNT/B, or Syt I_N_-Ab. Cells were subsequently permeabilized and immunostaining was carried out for the additional indicated intracellular proteins.

In Figure 5E, HEK293FT cells were growing on poly-D-lysine coated coverslips and were transfected with full-length SV2C in pCMV5 vector using Lipofectamine 2000 when cells reached 70–80% confluence. Cells were exposed to BoNT/D-HCR (80 nM) for 30 min at 37°C 48 hrs after the transfection. Cells were washed and fixed. Immunostaining was first carried out without permeabilization to detect BoNT/D-HCR. Subsequently, cells were permeabilized to detect SV2C using a polyclonal SV2C antibody. We note that the binding of BoNT/D-HCR was observed mostly in cells with round cell shapes – a morphology that becomes more prominent when cells reach complete confluence.

Functional entry of BoNTs into neurons was assayed by examining the cleavage of their substrate proteins in neurons. In general, neurons were exposed to indicated toxins for 5 min in high K^+^ buffers at 37°C. Neurons were washed, further incubated in toxin-free media for an additional 6 hrs, and lysed in the cell lysate buffer (PBS with 1% Triton X-100, 0.05% SDS and protease inhibitor cocktail (Roche, CA), 100 µl per one well of 24-well plates). Lysates were centrifuged for 10 min using a microcentrifuge at 4°C, and the supernatants were assayed by immunoblot analysis.

### Rapid-time-to-death assay and ganglioside loading

A rapid-time-to-death assay was utilized to assess the toxicity of BoNTs in GS KO mice, following a well-established protocol as previously described [57], [77]. Briefly, the WT and KO littermates were injected with the same amount of toxins intravenously (lateral tail vein), and their time-to-death was recorded. The apparent intraperitoneal LD_50_/ml of toxins in each mouse (effective toxicity) was determined using a standard curve as previously described [57], [77].

The stock of gangliosides was prepared by dissolving mixed bovine brain gangliosides in Chloroform:Methanol (2:1) solution. They were dried in glass tubes using nitrogen gas, resuspended in Neurobasal media at 1 mg/ml, and added to culture media at 250 µg/ml concentrations for 12 hrs to load into cell membranes.

## Supporting Information

Figure S1 SV2C expressed in a subpopulation of hippocampal neurons can mediate the binding of BoNT/D-HCR. Hippocampal neurons cultured from SV2A/B KO mice were exposed to BoNT/D-HCR (80 nM, 5 min in high K^+^ buffer). Cells were washed, fixed for immunostaining analysis using a polyclonal SV2C antibody and an anti-HA antibody. Binding of BoNT/D-HCR was observed to a subpopulation of synapses that express SV2C.
(EPS)

Figure S2 Chimeric receptor harboring the luminal domain of SV2 can mediate the binding of BoNT/A but not BoNT/D-HCR to SV2A/B KO neurons. SV2A/B KO neurons were transfected with a chimeric receptor containing the SV2A-L4-LDLR (upper panel) or SV2B-L4-LDLR (lower panel). Cells were exposed to either BoNT/A (20 nM) or BoNT/D-HCR (80 nM) for 20 min in media, washed and fixed for immunostaining analysis. Transfected cells were marked by co-expressed GFP. Expression of SV2A-LDLR or SV2B-L4-LDLR mediated the binding of BoNT/A to the cell surface, but failed to mediate the binding of BoNT/D-HCR.
(EPS)

Figure S3 The cytoplasmic domain of SV2C is not accessible to antibodies without permeabilizing cells. HEK293FT cells were transfected with SV2C. Cells were fixed and immunostaining was performed without permeabilizing cells for SV2C using a polyclonal SV2C antibody (SV2C poly Ab) that recognizes the N-terminal cytoplasmic domain of SV2C. Cells were subsequently permeabilized and immunostaining was carried out using a monoclonal SV2 antibody (SV2 mono Ab) that recognizes all SV2 isoforms. SV2C poly Ab failed to stain SV2C without permeabilizing cells, indicating that SV2C retains the correct topology on plasma membrane with its N-terminal domain resides inside cells.
(EPS)
